# Caregiver Satisfaction and Perceptions of Trauma-Focused Cognitive Behavioral Therapy

**DOI:** 10.1007/s40653-021-00372-y

**Published:** 2021-07-30

**Authors:** Lauren D. Brumley, Elisabeth Pollio, Beth Cooper, Robert A. Steer, Esther Deblinger

**Affiliations:** 1 CARES Institute, Rowan University School of Osteopathic Medicine, 42 East Laurel Road, Rowan Medicine Building, Suites 1100 & 1200, Stratford, NJ 08084 USA; 2 Department of Psychiatry, Rowan University School of Osteopathic Medicine, 42 East Laurel Road, Rowan Medicine Building, Stratford, NJ 08084 USA

**Keywords:** Caregivers, Treatment satisfaction, TF-CBT, Evidence-based treatment

## Abstract

Although caregivers have been found to be critical to children’s healing, little has been documented about caregivers’ experiences in Trauma-Focused Cognitive Behavioral Therapy (TF-CBT). The current study describes caregivers’ satisfaction with and perceptions of TF-CBT. Caregivers (*n* = 431) of children/adolescents (*n* = 496) who completed TF-CBT filled out pre-treatment questionnaires on demographics and perceived aloneness in facing their child’s trauma, and posttreatment questionnaires on treatment satisfaction and perceptions of TF-CBT. Caregivers rated treatment satisfaction an average of 30.59 (SD = 3.15) out of a maximum score of 32 on the Client Satisfaction Questionnaire-8. The majority of caregivers endorsed that talking about their child’s trauma was more helpful than discussing other current problems, they spoke frequently with their child’s therapist about their child’s trauma, they reported information/skill building in therapy were more helpful than support received, they felt understood by their therapist, treatment helped them more effectively parent, and treatment helped improve their relationship with their child. Perceptions were associated with overall treatment satisfaction. There was a significant reduction in caregivers’ feelings of aloneness in facing their child’s trauma from pre- to posttreatment, which was also related to overall treatment satisfaction. Caregivers reported high satisfaction with TF-CBT, and identified talking about their child’s trauma as more helpful than talking about problems not related to the trauma. Caregivers endorsed benefits of participating in TF-CBT, including feeling less alone in facing their child’s trauma, improved relationship with their child, and more effective parenting skills. These results have important treatment implications.

The efficacy and effectiveness of Trauma-Focused Cognitive Behavioral Therapy (TF-CBT) has been supported in numerous randomized controlled trials (e.g., Cohen et al., 2004; Cohen et al., 2011; Deblinger et al., 1996; Deblinger et al., 2011; Deblinger et al., 2006; Deblinger et al., 2001; Jensen et al., 2014). In addition, prior work has documented youth’s perceptions of TF-CBT (Cohen et al., 2006; Deblinger et al., 2011; Deblinger et al., 2006; Dittmann & Jensen, 2014); however, there is limited information in the literature about caregivers’ perceptions of their experience in TF-CBT. Caregivers are considered “gateway providers” to child mental health services (Stiffman et al., 2004) because they are key in determining whether youth initiate, attend, and complete treatment (Nock & Ferriter, 2005). It is important to understand what caregivers perceive as the benefits of participating in TF-CBT with their child to more optimally engage caregivers, and ultimately increase the likelihood that children receive and complete effective trauma-focused treatment. Thus, the current paper seeks to document caregiver satisfaction with TF-CBT as well as caregivers’ perceptions of TF-CBT.

Caregivers play an important role in the effectiveness of evidence-based treatments for children and adolescents (Dowell & Ogles, 2010; Karver et al., 2006). Regarding treatment for trauma in particular, children whose mothers were randomly assigned to participate in a trauma-focused evidence-based therapy condition for the child’s experience of sexual abuse exhibited greater reductions in behavioral and depressive symptoms compared to children who were randomly assigned to a therapy condition that did not involve the caregiver (Deblinger et al., 1996). In a randomized controlled trial comparing evidence-based trauma therapy to nondirective supportive therapy, parents’ emotional reaction to their child’s trauma was the strongest predictor of outcome, aside from treatment condition (Cohen & Mannarino, 1996). Caregiver support at follow-up in other studies was also associated with positive outcomes in preschool-aged children (Cohen & Mannarino, 1997) and school-aged youth (Yasinski et al., 2016). Consistent with those findings, lack of caregiver support, such as caregivers avoiding discussion of the trauma and/or blaming the child, was found to be associated with worsening internalizing and externalizing symptoms among youth (Yasinski et al., 2016). In addition, caregivers’ working alliance with their child’s therapist has been shown to increase over the course of TF-CBT and has also been associated with reductions in the child’s symptoms of posttraumatic stress (Loos et al., 2020). Together, this body of work highlights the critical role of caregivers in the effectiveness of trauma-focused therapy for youth.

Consumer satisfaction with mental health care is a multidimensional construct that includes client perceptions of the quality of services received, whether the consumer would recommend the services to others, and the clients’ subjective appraisal of change related to participation in the service (Fraser & Wu, 2016). Many studies have assessed consumer satisfaction using the well-established Client Satisfaction Questionnaire-8 (CSQ-8; Attkisson, 2020; Larsen et al., 1979). Studies of community-based outpatient mental health treatment (‘treatment as usual’) for youth have reported that caregivers rate items on the CSQ-8 an average of 3.19 to 3.45 on the 4-point scale (Garland et al., 2007; Godley et al., 1998; Kapp et al., 2017), which translates to an approximate total score of 25.52 to 27.60 out of the maximum total score of 32. According to a pilot study of TF-CBT for traumatic grief, caregivers rated consumer satisfaction on the CSQ-8 an average of 29.69 out of maximum possible score of 32 (Cohen et al., 2006). This translates to an item mean score of approximately 3.71 on the 4-point scale. Thus, although it is not possible to draw inferential conclusions regarding whether there is a statistically meaningful difference between CSQ-8 ratings of TF-CBT versus treatment as usual in these studies, it appears that caregivers generally provide very high satisfaction ratings for TF-CBT. Additionally, it is important to examine caregiver satisfaction with TF-CBT in a broader sample among caregivers of youth who have experienced traumas beyond traumatic grief.

The literature also recommends exploring what specific treatment elements clients feel most satisfied with as well as which elements of treatment they prefer (Fraser & Wu, 2016). Several studies have reported that most children (Deblinger et al., 2011) and adolescents (Deblinger et al., 2006; Dittmann & Jensen, 2014) identify talking about the trauma as the most helpful aspect of TF-CBT. In addition, one recent qualitative study reported that youth who completed the trauma narrative described the importance of caregiver involvement in their treatment and thought this involvement improved their family communication (Okamura et al., 2020). These findings are particularly interesting given that some caregivers do not initiate trauma-focused therapy for their children due to concerns that talking about the trauma in therapy will be re-traumatizing (Fong et al., 2016). Previous studies, however, did not report what participating caregivers identified as being the most helpful aspect of TF-CBT nor did they examine whether caregivers’ perceptions of talking about their child’s traumatic experiences during the course of TF-CBT was related to treatment satisfaction.

Caregivers who are more involved in their child’s outpatient mental health treatment tend to report having a more positive experience in treatment (Holmboe et al., 2011). Caregiver involvement in treatment may be particularly significant in the context of trauma therapy given evidence that following a child’s disclosure of abuse, nonoffending caregivers often experience significant feelings of guilt, distress, and isolation (Deblinger et al., 1994; Fong et al., 2020), and some caregivers experience worsening family relationships and loss of support systems (Fong et al., 2020). Furthermore, mothers who have less social support (Hiebert-Murphy, 1998) or feel more alone (Deblinger et al., 1993) following their child’s disclosure of sexual abuse are more emotionally distressed than mothers with more social support. It has been theorized that it is therapeutic for caregivers to be involved in their child’s treatment to increase feelings of support, while also enhancing coping and parenting skills designed to positively influence their child’s wellbeing (Deblinger et al., 2015). For this and many other reasons, TF-CBT includes a high degree of caregiver involvement throughout treatment (Cohen et al., 2017; Deblinger et al., 2015). Interestingly, there is some evidence that caregivers who report greater parenting stress are more likely to complete TF-CBT compared to caregivers with lower parenting stress (Lai et al., 2019). It is unknown whether caregivers’ perceptions of feeling alone change over the course of participating in TF-CBT, and if this relates to their satisfaction with therapy.

The present study is the first to our knowledge to examine caregiver satisfaction with and perceptions of TF-CBT for various trauma types. First, we sought to document caregivers’ overall satisfaction with TF-CBT and their perceptions of TF-CBT (e.g., the most helpful aspect of TF-CBT, feeling alone in facing the trauma, how often the caregiver talked with therapist about child’s trauma). Consistent with the pilot study of TF-CBT for traumatic grief (Cohen et al., 2006), it is hypothesized that caregivers participating with children impacted by diverse traumas will report very high satisfaction with TF-CBT on a standardized measure. We also expected that, like children and adolescents (Deblinger et al., 2011; Deblinger et al., 2006; Dittmann & Jensen, 2014), most caregivers will endorse talking about the trauma as the most helpful aspect of TF-CBT as compared to talking about current problems not related to the trauma. In addition, we hypothesized that the majority of caregivers would endorse feeling understood by their child’s therapist, that they talked frequently with the therapist about their child’s trauma, and that therapy improved parenting skills and the caregiver-child relationship. We expected that these perceptions would relate to overall caregiver satisfaction when included in a single regression model. We also expected that feelings of aloneness in facing their child’s trauma would decrease from pre- to posttreatment, and that this would relate to greater satisfaction with treatment.

## Method

The current sample included 431 non-offending caregivers of 496 youth who completed TF-CBT between March 2004 and April 2020 at a regional diagnostic and treatment center that specializes in the assessment and treatment of child abuse/trauma. After providing treatment consent but prior to the administration of pre-treatment assessment measures, all families who receive therapy services at the clinic are invited to participate in research. They are asked to allow their responses to standardized assessment measures and semi-structured interviews to be entered into a research repository for use in possible future research. Potential participants are reminded that allowing use of their responses for research purposes is voluntary and declining to participate would not impact the services provided. Caregivers and youth ages 14 to 21 years provide written research consent, whereas children ages 7 to 13 years provide research assent. Caregivers and youth then complete questionnaires and a semi-structured interview as part of routine clinical care before and after completing therapy. Only responses from those caregivers and children who provided research consent/assent and met the following inclusionary criteria were included in the sample: 1) youth completed TF-CBT, 2) a non-offending caregiver (i.e., a caregiver who did not perpetrate the abuse for which the child was in treatment) participated in TF-CBT with the youth, 3) the caregiver completed the CSQ-8, and 4) questionnaires were completed in English. The above outlined procedures as well as the current study were approved by the Institutional Review Board at the medical school where the research took place.

In the current sample, youth receiving TF-CBT were an average of 9.11 years old (*SD* = 3.72, 3–18 years old). Per caregiver-report, 67% of youth were female, 51% White, 23% Black or African American, 11% biracial, and 14% other race. Thirty-four percent of the youth in the sample were Hispanic. The most common reasons for referral to TF-CBT included a ‘not okay touch’ by another child (34%), child sexual abuse by an adult caregiver (27%) or adult non-caregiver (24%), physical abuse (by someone other than the participating caregiver, 6%), exposure to domestic violence (3%), or other trauma (5%). The majority of participating caregivers were biological or adoptive parents (84%) and identified as female (89%). Sixty percent of caregivers identified as White (60%), 21% Black or African American, 3% biracial, and 16% other race. Twenty-one percent of caregivers were Hispanic. Caregivers were between 21 and 72 years old (*M* = 37.82, *SD* = 9.43). Approximately half of caregivers in the sample were married or living with a partner (46%). Regarding highest level of education attained, 12% of caregivers did not complete high school, 43% completed high school, 24% had an associate’s degree or technical schooling, 10% had a bachelor’s degree, and 1.5% completed graduate school. Of note, 10% of caregivers preferred not to answer the question on educational status. Fifty-nine percent of caregivers were employed at the time of treatment. See Table 1 for more details on sample characteristics.

**Table 1:** Sample characteristics (*n* = 431 caregivers of 496 youth)

Variable	*n* (%) or *M* (*SD*), range
Youth Service Entry Characteristics (*n* = 496)	
Youth age	9.11 (3.72), 3–18
Youth gender (female)	331 (67%)
Youth race	
White/Caucasian	253 (51%)
Black or African American	113 (23%)
American Indian or Alaska Native	0
Asian American	1 (0.2%)
Biracial	55 (11%)
Other	71 (14%)
Youth ethnicity	
Non-Hispanic	324 (65%)
Hispanic	93 (19%)
Reason for referral to TF-CBT	
Child sexual abuse by caregiver	136 (27%)
Child sexual abuse by non-caregiver	121 (24%)
Child physical abuse	28 (6%)
Inappropriate sexual contact initiated by another child	169 (34%)
Inappropriate physical contact initiated by another child	20 (4%)
Child witnessed domestic violence	14 (3%)
Other traumatic events	25 (5%)
Caregiver Service Entry Characteristics (*n* = 431)	
Caregiver relationship to child (biological/adoptive parent)	363 (84%)
Caregiver age	37.82 (9.43), 21–79
Caregiver gender (female)	383 (89%)
Caregiver married or living with partner	198 (46%)
Caregiver race	
White/Caucasian	259 (60%)
Black or African American	90 (21%)
American Indian or Alaska Native	1 (0.2%)
Asian American	1 (0.2%)
Biracial	11 (3%)
Other	61 (14%)
Caregiver ethnicity	
Non-Hispanic	342 (79%)
Hispanic	72 (17%)
Caregiver highest level of education	
Did not complete high school	52 (12%)
Completed high school	185 (43%)
Associate’s Degree or Technical School	103 (24%)
Bachelor’s Degree	42 (10%)
Master’s Degree	5 (1%)
Doctoral Degree	2 (0.5%)
Prefer not to answer	42 (10%)
Employment Status	
Not currently employed	137 (32%)
Employed	254 (59%)

Percentages may not add to 100% due to rounding or missing data

### Measures

#### Demographics and Background Information

At the initial treatment session, caregivers reported their and their child’s age, gender identity, race, and ethnicity. In the early years of data collection, race/ethnicity was asked as one item, and later race and ethnicity were split into two separate items to be consistent with National Institutes of Health (NIH) standards. Thus, caregivers who selected Hispanic as a race in the original item were recoded as “Other” for race and Hispanic/Latino for ethnicity. Caregivers also self-reported their relationship with the child (e.g., biological parent, adoptive parent, grandparent, foster parent), marital/partnered status, highest level of education attained, and employment status. Therapists completed an interview with caregivers at the initial treatment session during which they recorded the reason for referral to TF-CBT (e.g., sexual abuse, physical abuse, witnessing domestic violence) reported by the caregiver and in records provided from the referral source (such as the child protection agency or Prosecutor’s office).

#### Client Satisfaction Questionniare-8© (CSQ-8©)

The CSQ-8 is a self-report instrument designed to measure satisfaction with care (Attkisson, 2020; Larsen et al., 1979). It is composed of eight items that are rated on different 4-point Likert scales, such as “How would you rate the quality of service you received?” (1 = *poor*, 4 = *excellent*) and “Have the services you received helped you to deal more effectively with your problem?” (1 = *No, they seemed to make things worse*, 4 = *Yes, they helped a great deal*). Responses are summed to form a total score ranging from 8 to 32, with higher values representing greater satisfaction with services. The CSQ-8 is widely-used in mental health services research (Fraser & Wu, 2016), and had very good internal consistency in this sample (α = .89).

#### Supplemental Items Assessing Caregiver Perceptions of Treatment

These questions are part of the clinical interview designed by the regional diagnostic and treatment center at which the study took place.

##### Perception of Facing the Child’s Trauma Alone

At pre- and posttreatment, caregivers rated the support they have/had in helping them deal with their child’s trauma using the following item: “To what extent do you feel you are facing this alone?” (1 = *not at all*, 2 = *to some extent*, 3 = *to a great extent*).

##### Perception of “Most Helpful” Aspects of Treatment

On a posttreatment questionnaire, caregivers responded to the following item: “Which of these two aspects of therapy was most helpful?” (1 = *talking about [the child’s trauma]*, 2 = *talking about current problems and concerns not related to [the child’s trauma]*). Before providing the questionnaire to the caregiver, the therapist or another trained assessor filled in a blank space with the child’s trauma (e.g., sexual abuse, physical abuse, placement in foster care). Caregivers responded to another item asking: “Which of these two aspects of therapy was most helpful?” using the following choices: 1 = *the support you received*, 2 = *the information/skills you received*.

##### Perception of Feeling Understood by the Therapist

At posttreatment, caregivers rated the extent to which they felt understood (“Do you think your counselor understood your feelings and problems?”) using a scale of 1 = *yes, all the time* to 4 = *No.* Responses were reverse-scored so that higher ratings reflected feeling more understood.

##### Perceptions of Trauma-Focused Versus Other Content of Therapy Sessions

At posttreatment, caregivers were asked to rate the following item using a scale of 1 = *not at all* to 4 = *frequently:* “How often did you talk with the therapist about your child’s experience of [trauma]?”

##### Perceptions of Impact of Treatment on Parenting and Child-Caregiver Relationship

Caregivers rated (1 = *No, they seemed to make things worse,* 4 = *Yes, they helped a great deal*) the extent to which services “helped you effectively parent your child” and “helped improve you and your child’s relationship.”

### Data Analysis Plan

Analyses were performed with SPSS Version 27. Frequencies and descriptive statistics were performed for all variables. There was less than 6% missing data on perception variables measured at posttreatment. Eighteen percent of data on pre-treatment aloneness was missing due to a variety of random factors, such as the question being omitted on an early iteration of the pre-treatment interview and therapists inadvertently skipping the question.

Correlations were performed among caregiver perception variables and CSQ-8 total score. Pearson product-moment correlations were performed to examine the associations between the continuous perception variables and the CSQ-8 total score, and point-biserial correlations were performed to examine the associations between binary perception variables and the CSQ-8. The significance level (two-tailed test) was set by using a Bonferroni adjustment of 0.05/8 (*p* < .006) to control for the familywise error rate for these correlations. A paired-samples t-test was performed to assess whether caregivers’ ratings of perceived aloneness in facing the child’s trauma changed from pre- to posttreatment.

Two ordinary least squares (OLS) regression analyses were calculated to estimate caregiver satisfaction (CSQ-8 total score) from perceptions. We first determined whether the CSQ-8 or any perception variables were significantly correlated with any demographic variables (i.e., the child’s age, sex, or racial/ethnic minority status; the caregiver’s age, sex, or racial/ethnic minority status, relationship to the child, marital/partnered status, educational status, employment status) and also had at least medium effect sizes, i.e., *r* > .30 (Cohen, 1992). These correlations were performed to determine whether any of these demographic characteristics might need to be controlled for in the regression estimating the CSQ-8 total score. The first OLS regression was performed to estimate caregiver satisfaction (CSQ-8) from caregiver perception variables that were significantly correlated with the CSQ-8, as well as any significant demographic correlates. The second OLS regression was performed to estimate treatment satisfaction (CSQ-8) with respect to posttreatment feelings of aloneness, controlling for pre-treatment feelings of aloneness and any significant demographic correlates. Because there was 22% missing data for these variables, the OLS regression analysis was restricted to only caregivers with pre- and posttreatment perceived aloneness data (*n* = 338).

## Results

### Caregiver Treatment Satisfaction and Perceptions

Table 2 presents an overview of frequencies and descriptive statistics for caregiver satisfaction and perceptions reported at posttreatment. Overall, caregivers provided high ratings of treatment satisfaction (*M* = 30.59, *SD* = 3.15) on the CSQ-8, which has a maximum total score of 32. The majority (68%) of caregivers endorsed talking about the trauma as more helpful than talking about other current problems not related to the trauma. Eighty-two percent of caregivers indicated that they “frequently” talked with their therapist about their child’s experience of trauma. A slight majority of caregivers found the information/skills received (55%) to be more helpful than the support they received during therapy. Caregivers indicated feeling understood by the therapist, as 89% endorsed that the therapist understood their feelings and problems “all the time.” Seventy-one percent of caregivers indicated that treatment helped “a great deal” with their ability to effectively parent their child, and 71% of caregivers thought that treatment had helped “a great deal” with improving their relationship with their child.

**Table 2:** Caregiver satisfaction and perceptions at posttreatment (*n* = 431)

Variable	*n* (%) or *M* (*SD*), range
CSQ-8 Total Score	30.59 (3.15), 8–32
Perception of most helpful aspect of treatment	
Talking about the trauma	291 (68%)
Talking about other problems (not trauma-related)	97 (23%)
Perception of most helpful aspect of treatment	
The support you received	146 (34%)
The information/skills you received	235 (55%)
Perception that therapist understood your feelings and problems	
No	1 (0.2%)
Sometimes	4 (1%)
Yes, most of the time	43 (10%)
Yes, all the time	383 (89%)
Perception of how often you talked with the therapist about your child’s experience of trauma	
Not at all	1 (0.2%)
Rarely	11 (3%)
Sometimes	58 (14%)
Frequently	354 (82%)
Perception that the services helped you effectively parent your child	
No, they seemed to make things worse	0
No, they really didn’t help	7 (2%)
Yes, they helped somewhat	110 (23%)
Yes, they helped a great deal	338 (71%)
Perception that services helped improve you and your child’s relationship	3.73 (0.47), 2–4
No, they seemed to make things worse	0
No, they really didn’t help	6 (1%)
Yes, they helped somewhat	93 (22%)
Yes, they helped a great deal	307 (71%)

Percentages may not add to 100% due to rounding or missing data

### Caregiver Perceptions Correlated with Overall Satisfaction

First, as the correlations in Table 3 show, the caregivers who were more satisfied with treatment overall felt their therapist understood their problems and feelings (*r* = .42, *p* < .001), reported talking more frequently about the trauma with the child’s therapist (*r* = .33, *p* < .001), indicated the services provided helped them more effectively parent their child (*r* = .43, *p* < .001), and thought the services helped to improve their relationship with their child (*r* = .31, *p* < .001).

**Table 3:** Correlations of perceptions of TF-CBT with caregiver satisfaction

	n	Caregiver Satisfaction (CSQ-8)
Feel you are facing this alone (Pre-treatment)	354	.02
Feel you are facing this alone (Posttreatment)	410	−.17*
Most helpful part of treatment support (0) or skills (1)	381	−.01
Most helpful part of treatment talking about other problems (0) or trauma (1)	388	.10
Felt understood by child’s therapist	431	.42*
How often talked with therapist about child’s trauma	424	.33*
Services helped you effectively parent	410	.43*
Services improved your and child’s relationship	406	.31*

*p < .006 using a Bonferroni adjustment of alpha/8 to control for the familywise error rate

Next, we tested which perceptions were associated with overall caregiver satisfaction (CSQ-8) when included in a single regression model. None of the caregiver or child demographic variables were significantly correlated with any of the perceptions or CSQ-8 at the bivariate level at *r* > .30. Therefore, no demographic variables were included as control variables in the regression model. As shown in Table 4, caregivers who felt more understood by their child’s therapist, talked more often with the therapist about the child’s trauma, and thought that treatment helped them more effectively parent were more satisfied with TF-CBT overall. Thinking that the caregiver-child relationship improved over treatment was not significant in the regression model. Overall, these caregiver perceptions explained approximately 44% of the variance in caregiver satisfaction.

**Table 4:** OLS regression predicting caregiver satisfaction from caregiver perceptions (*n* = 401)

	Caregiver satisfaction (CSQ-8)		
Predictor	*b* (*SE*)	β	*p*
Felt understood by child’s therapist	2.52 (0.24)	0.42	*< .001*
How often talked with therapist about child’s trauma	0.81 (0.19)	0.17	*< .001*
Services helped you effectively parent	1.36 (0.24)	0.29	*< .001*
Services improved your and child’s relationship	0.12 (0.24)	0.03	.62
Model R^2^	.44		

The model includes caregiver perceptions that were significantly associated with CSQ-8 in bivariate correlations. Two-tail significance level used a Bonferroni adjustment of alpha/4 (*p* < .01) to control for the familywise error rate

### Feeling Alone in Facing the Child’s Trauma

Caregivers reported significantly reduced feelings of aloneness in dealing with their child’s trauma at posttreatment (*M* = 1.44, *SD* = 0.70) compared to pre-treatment (*M* = 1.82, *SD* = 0.82; paired *t*(373) = 7.70, *p* < .001). At the beginning of treatment, 21% of caregivers endorsed feeling alone “to a great extent” in facing their child’s trauma. However, by the end of treatment, only 11% of caregivers endorsed feeling alone “to a great extent.” The percent of caregivers who felt “not at all” alone in facing their child’s trauma almost doubled from pre-treatment (36%) to posttreatment (63%).

None of the caregiver or child demographics were significantly correlated with pre- or posttreatment aloneness or CSQ-8 with a medium effect size (*r* > .30; Cohen, 1992); therefore, these demographic variables were not controlled for in the regression. As shown in Table 5, controlling for feelings of aloneness at pre-treatment, caregivers who felt less alone at posttreatment were more satisfied with treatment (*b* = −0.78, *SE* = .22, β = −0.19, *p* = .001), which explained approximately 3.7% of the variance in treatment satisfaction.

**Table 5:** OLS regression predicting caregiver satisfaction from caregiver perceived aloneness (*n* = 338)

	Caregiver Satisfaction (CSQ-8)		
Predictor	*b* (*SE*)	β	*p*
Feel you are facing this alone (Pre-treatment)	0.26 (0.19)	0.08	.16
Feel you are facing this alone (Posttreatment)	−0.78 (0.22)	−0.19	.*001*
Model R^2^	.04		

Two-tail significance level used a Bonferroni adjustment of alpha/2 (*p* < .03) to control for the familywise error rate

## Discussion

This is the first study to our knowledge to examine treatment satisfaction and perceptions among a large group of caregivers participating in TF-CBT with children who have experienced various forms of trauma such as sexual abuse, inappropriate sexual contact initiated by another child, physical abuse, exposure to domestic violence, and/or other trauma (e.g., traumatic grief, neglect). Consistent with our hypotheses, caregivers were highly satisfied with TF-CBT overall, replicating the findings of an earlier study examining consumer satisfaction among caregivers whose children were impacted by traumatic loss (Cohen et al., 2006). In addition, as expected, caregivers participating in this study reported feeling understood by their therapist, and thought treatment enhanced effective parenting and improved their relationship with their child, which is also consistent with prior findings that TF-CBT improves parenting skills (Cohen et al., 2004; Deblinger et al., 1996) and enhances children’s feelings of relatedness (Deblinger et al., 2017). Skills received in treatment were perceived as most helpful by more caregivers as compared to support received.

Through gradual exposure, TF-CBT directly addresses the traumatic experience(s) and therefore it is not surprising that the vast majority of caregivers indicated they spoke frequently with the therapist about their child’s trauma. Although caregivers and some new TF-CBT therapists may initially feel uncomfortable directly discussing trauma, it is important to note that caregivers who indicated they spoke more frequently about the trauma were significantly more satisfied with treatment. In addition, overall treatment satisfaction was positively related to caregivers’ feelings that treatment had enhanced their parenting skills and their relationship with their child. When perceptions were examined together in a regression model, feeling understood by their child’s therapist, talking more often with the therapist about their child’s trauma, and thinking that services helped them more effectively parent were perceptions accounted for a significant portion of variance in overall caregiver satisfaction. In other words, these results demonstrate that caregivers feel more satisfied when they feel understood by the therapist, talk with the therapist about their child’s trauma, and receive guidance on parenting. Caregivers’ perceptions that the relationship with their child was enhanced did not significantly contribute additional unique variance in the regression model. This may be due to this perception item possibly being correlated with feelings of enhanced parenting skills. In sum, these findings reinforce several central tenets of TF-CBT concerning the importance of the therapeutic relationship, the value of openly discussing the trauma(s), and the focus on enhancing parenting.

Further, caregivers felt significantly less alone in facing the child’s trauma from pre- to posttreatment, also possibly reflecting the assistance they received from the therapist. The change in feeling alone explained a significant proportion of the variance in overall satisfaction. Not surprisingly, caregivers who experienced greater reductions in feeling alone at posttreatment were more satisfied with therapy. Once again, attention to the therapist-client relationship appears to be an important part of the experience of caregivers participating in TF-CBT. It is important to note that due to the nature of correlational data, we cannot rule out the possibility that this positive change in perceived support could be attributable to other factors, such as the passage of time or sources of support outside of TF-CBT, such as if the caregiver participated in their own therapy in addition to TF-CBT for their child. Although we are unable to report the exact percent of caregivers participating in their own therapy while participating in TF-CBT for the subset of caregivers in the current study, we have found based on prior clinical and research experience that a only very small percent of caregivers participate in individual therapy with another therapist during their participation in TF- CBT with their child.

These results have several implications for treatment. Although evidence-based treatments have at times been criticized for placing less emphasis on the therapist-client relationship, these results clearly indicate that caregivers participating in TF-CBT were highly satisfied with treatment, felt understood by their therapist, and felt significantly less alone in facing their child’s trauma by the end of treatment possibly reflecting the emphasis placed on the therapeutic relationship in the context of TF-CBT. In fact, other recent research findings have documented the positive influence of a strong therapeutic alliance with youth on TF-CBT outcomes (Ormhaug et al., 2014) as well as on caregivers’ willingness to engage in TF-CBT (Okamura et al., 2020). In addition, when asked if support or skills received was the most helpful aspect of treatment, skills received was identified as most helpful by more caregivers, which supports the utility of teaching skills as part of treatment. Further, the finding that treatment satisfaction is related to enhancing effective parenting skills and improving the child-caregiver relationship supports TF-CBT’s emphasis on these aspects of treatment. Finally, although directly discussing the trauma in treatment can potentially be a source of discomfort initially, the findings from the present study indicate that talking frequently about the trauma is related to caregiver satisfaction with treatment. In sum, the results suggest that it is beneficial for therapists to directly address the trauma, teach skills as a part of treatment, including effective coping and parenting skills, and help to enhance the caregiver-child relationship.

Some child therapies, including trauma-focused therapies, engage caregivers very informally or not at all. The findings from the present study suggest that the recommended practice of spending a significant portion of each TF-CBT session with caregivers may reduce the caregivers’ feelings of aloneness and enhance their satisfaction with treatment overall. Thus, along with noting the effectiveness of TF-CBT on ameliorating children’s symptoms and improving their resiliency, the current findings may also be used to help effectively engage caregivers in treatment, particularly those caregivers who may be reluctant to participate in a treatment that directly addresses their child’s trauma. Helping caregivers understand that they will be actively involved in treatment and will play a critical role in helping their child heal may be important to motivate caregivers to fully engage in and complete treatment with their child.

The current study, like most investigations, does have its limitations. The first and most significant one is the lack of a comparison condition. It is, therefore, not possible to directly compare caregiver satisfaction with TF-CBT to other trauma-focused therapies for children. Also, given the generally descriptive and correlational nature of the data set, the results should be interpreted with caution. The findings reflect associations between variables rather than predictive relationships, and in some cases, positive change in caregivers’ perceptions could be attributable to other factors rather than to TF-CBT. In addition, while the CSQ-8 is a well-established measure, the supplemental questions regarding caregivers’ perceptions of their treatment experiences have not been normed or evaluated in terms of their psychometric properties. Examining the reliability and content validity of these supplemental items will be important to strengthen the basis for their inclusion in future research. The sample represents caregivers who agreed to allow their and their children’s responses to be used for future research, and therefore the findings may not be generalizable to all caregivers accompanying their children to treatment in terms of attitudes toward research and possibly evidence-based treatment. We were unable to examine the impact of socioeconomic status on caregiver satisfaction and perceptions of TF-CBT as we did not have a measure of socioeconomic status available in this data set. Our measure of caregiver educational and employment status only captured the status of the participating caregiver, not the highest educational or employment status of any adult in the home. Finally, this study did not include symptom outcome measures. However, it should be noted that treatment satisfaction has not necessarily been found to be associated with objective treatment outcomes (Garland et al., 2003; Turchik et al., 2010).

Future researchers are encouraged to include and report on consumer satisfaction measures in randomized trials so that we can better understand not only the efficacy of various trauma-focused therapies, but also the relative levels of satisfaction that clients report. Results highlight the importance of asking supplemental questions on clients’ perceptions of therapy in addition to general consumer satisfaction, such as perceptions of feeling understood by the therapist and talking with the therapist about the trauma. Furthermore, it should be noted that the current investigation included only caregivers who completed treatment. Future investigators might attempt to obtain client satisfaction data from those clients who do not complete treatment. Such data could be challenging to obtain, but might provide insight about what factors led to premature withdrawal from treatment. Qualitative research in which clients are invited to share their thoughts and feelings about therapy in open-ended narratives or interviews might also be of great value in learning about clients’ perspectives of what seems to work and not work in terms of treatment engagement and completion. It would also be important in future research to gather information on families’ socioeconomic status to examine whether caregiver satisfaction varies as a function of SES. Finally, this investigation focused on caregiver satisfaction, but more comprehensive research on children’s levels of consumer satisfaction and their perspectives on treatment would also be a valuable addition to the literature.
